# Isolation and Molecular Characterization of the Romaine Lettuce Phylloplane Mycobiome

**DOI:** 10.3390/jof7040277

**Published:** 2021-04-07

**Authors:** Danny Haelewaters, Hector Urbina, Samuel Brown, Shannon Newerth-Henson, M. Catherine Aime

**Affiliations:** 1 Department of Botany and Plant Pathology, Purdue University, West Lafayette, IN 47907, USA; hurbinay@gmail.com (H.U.); sambrown1119@gmail.com (S.B.); newerthshannon@gmail.com (S.N.-H.); 2 Division of Plant Industry, Florida Department of Agriculture and Consumer Services, Gainesville, FL 32608, USA

**Keywords:** fungal community, fungal diversity, phylloplane, species identification, Sporidiobolales, yeasts

## Abstract

Romaine lettuce (*Lactuca sativa*) is an important staple of American agriculture. Unlike many vegetables, romaine lettuce is typically consumed raw. Phylloplane microbes occur naturally on plant leaves; consumption of uncooked leaves includes consumption of phylloplane microbes. Despite this fact, the microbes that naturally occur on produce such as romaine lettuce are for the most part uncharacterized. In this study, we conducted culture-based studies of the fungal romaine lettuce phylloplane community from organic and conventionally grown samples. In addition to an enumeration of all such microbes, we define and provide a discussion of the genera that form the “core” romaine lettuce mycobiome, which represent 85.5% of all obtained isolates: *Alternaria*, *Aureobasidium*, *Cladosporium*, *Filobasidium*, *Naganishia*, *Papiliotrema*, *Rhodotorula*, *Sampaiozyma*, *Sporobolomyces*, *Symmetrospora* and *Vishniacozyma*. We highlight the need for additional mycological expertise in that 23% of species in these core genera appear to be new to science and resolve some taxonomic issues we encountered during our work with new combinations for *Aureobasidium* *bupleuri* and *Curvibasidium nothofagi*. Finally, our work lays the ground for future studies that seek to understand the effect these communities may have on preventing or facilitating establishment of exogenous microbes, such as food spoilage microbes and plant or human pathogens.

## 1. Introduction

Microorganisms—bacteria and fungi, including yeasts—are ubiquitous components of the food supply. The rise in organic food production has resulted in, among other things, an increase in naturally occurring microbial populations on produce. Whereas the majority of microbiota on fresh produce are likely to be harmless or even beneficial, many species are responsible for food spoilage and some can cause serious diseases in humans [1]. For example, the *Fusarium fujikuroi* Nirenberg species complex (FFSC) encompasses over fifty phylogenetic species, a number of which were found to be among the principal fungal contaminants of maize grains from rural commodity markets in Kenya [2]. These species are causal agents for ear rot of maize and all of them produce fumonisins and related mycotoxins that are carcinogenic in humans and animals [3,4].

The effect that natural plant microbial communities may have on exogenous (=introduced) microbes (such as plant or human pathogens) is poorly understood, to the extent that basic data are lacking as to whether microbial interactions may inhibit or facilitate pathogen spread. In addition, for most produce, essential basic data on naturally occurring microbial communities are missing.

Romaine lettuce (*Lactuca sativa* L.) is one of the most commonly used vegetable crops in the USA, with a rapidly expanding market [5]. The romaine lettuce leaf surface, or phylloplane, like that of all plant leaves, is home to innumerable microbes—fungal and bacterial. However, because lettuce leaves are nearly always consumed raw rather than cooked, the microbes on the lettuce phylloplane are often ingested by the consumer. Many outbreaks of human pathogenic bacteria, such as shiga-toxin producing *Escherichia coli*, are associated with romaine lettuce; it has been among the fresh produce items most implicated in food-borne diseases between 1973 and 1997 [6]. *Escherichia coli* strain O157:H7 is the most common bacterial causal agent of the outbreaks associated with romaine lettuce and other leafy green vegetables [7,8,9,10]. Sources of *E. coli* outbreaks on leafy greens such as lettuce may be contaminated soil, irrigated water and fecal animal deposits [11,12,13]. During the mid-2018 outbreak, 210 people from 36 states were infected with *E. coli* O157:H7; 96 were hospitalized. This *E. coli* strain can cause bloody diarrhea, stomach cramps, vomiting and kidney failure. In some cases, the gut poisoning can be fatal; following the mid-2018 outbreak, five people died [14].

Despite this, our knowledge about natural fungal species associated with the phylloplane of romaine lettuce is still limited, yet this knowledge is necessary for understanding how this naturally occurring flora interacts with introduced human pathogens on the phylloplane. In a large-scale effort to characterize the fungal microbiota of romaine lettuce from a public health perspective, we want to answer the following questions: (i) What species are typically part of the fungal community on romaine lettuce leaves? (ii) What drives changes in fungal communities over time? (iii) Which romaine lettuce community type—organic versus conventional—is most vulnerable to establishment by *E. coli*? Resolving these questions is critical to understanding the ability of human-pathogenic bacteria to enter and persist in these romaine lettuce fungal communities as well to developing a reasonable strategy for mitigating against future outbreaks of *E. coli*.

Toward that goal, in this paper, we investigate the fungal communities present on the phylloplane of commercially grown romaine lettuce obtained bought in grocery stores in the USA, as well as lettuce heads grown hydroponically. We combined culturing techniques and a Sanger sequencing approach to compare communities between conventionally grown and organic samples.

## 2. Materials and Methods

### 2.1. Collection of Samples and Fungal Isolation

Lettuce heads were obtained from commercial vendors in Indiana (Indianapolis, Lafayette, West Lafayette), Illinois (Champaign, Chicago), Virginia (Springfield) and Washington, D.C. between April and December, 2016. In addition, we obtained three hydroponically grown romaine lettuce heads from an indoor environmentally controlled facility based in Cleveland, Ohio. Lettuce heads were stored at 4 °C overnight and then immediately processed. In total, we examined 42 lettuce heads—grown either conventionally (*n* = 25), organically (*n* = 14), or hydroponically (*n* = 3). External leaves were discarded prior to random sampling of leaves from the exterior towards the center of each lettuce head. A sample of 25 g from different leaves was obtained from each lettuce plant and blended in 225 mL of sterile 100 µM phosphate buffer (5.4 g/L monosodium phosphate + 8.7 g/L disodium phosphate). From each sample, a 1 mL aliquot was plated in serial dilutions up to 10-3 on each of the following media to recover different groups of fungi (yeasts, filamentous and slow-growing): (i) Yeast Malt extract Agar (YMA), (ii) Potato Dextrose Agar (PDA) and (iii) Rose Bengal Agar (RBA), all with 2% agar and supplemented with 50 µg/mL chloramphenicol and 100 µg/mL ampicillin (all BD, Franklin Lakes, NJ, USA) to inhibit bacterial growth.

From each lettuce sample, up to 12 colonies were chosen for subculturing. Selection of colonies was based on morphology; colonies with differing morphologies on each plate were selected; additional colonies with similar morphologies were selected secondarily to increase the likelihood of obtaining morphologically similar but phylogenetically different species. Axenic strains obtained after several rounds of subculturing are maintained on PDA slants at 4 °C and in 15% (for filamentous strains) or 40% (for yeast-like strains) glycerol at −80 °C in the culture collection of M.C. Aime (MCA), Department of Botany and Plant Pathology, Purdue University, West Lafayette, IN, USA.

### 2.2. Identification of Fungal Isolates

DNA from axenic cultures was extracted using the Wizard Genomic DNA Purification Kit (Promega, Madison, Wisconsin) or amplified directly using a colony PCR method [15]. We amplified the internal transcribed spacer region of the ribosomal DNA (ITS) for all pure strains. Primer combinations used were ITS1f/ITS4 and ITS1f/ITS4B [16,17]. Amplifications were performed on a pro S Mastercycler (Eppendorf, Hauppauge, New York) in 25 reactions containing 12.5 μL of 2× MyTaq Mix (Bioline, Swedesboro, New Jersey), 9.5 µL of ddH_2_O and 1.0 µL of DNA. Cycling conditions included an initial denaturation at 94 °C for 5 min; followed by 35 cycles of denaturation at 94 °C for 30 s, annealing at 50 °C for 45 s, extension at 72 °C for 45 s; and a final extension step at 72 °C for 7 min. PCR products were visualized on 1% agarose gel using 1× TAE buffer and SYBR GelRed. Purification and sequencing were outsourced to Genewiz (Plainfield, NJ, USA). Sequence reads were assembled, trimmed and edited in Sequencher 5.0 (Gene Codes Corporation, Ann Arbor, Michigan). All forward and reverse identical reads were collapsed to separate unique sequences. Generated sequences were deposited to NCBI GenBank (accession numbers in Supplementary Table S1). All ITS sequences were subjected to a nucleotide BLAST search for initial identification to genus level (http://blast.ncbi.nlm.nih.gov/Blast.cgi, accessed on 7 April 2021). Tentative generic IDs were confirmed by morphology of originating cultures.

### 2.3. Statistical Analyses

To determine whether treatment (conventional, organic, hydroponic) had a significant effect on the presence of fungal isolates, we performed a one-way ANOVA with α = 0.05. Because the three-treatment dataset was biased (30 isolates from hydroponic samples versus 147 from conventional samples and 154 from organic samples), we also analyzed the effect of treatment only considering fungal isolates from conventional and organic lettuce samples. We calculated the sample variances for both conventional and organic isolates (25.36 and 26.11, respectively) and then performed a two-tailed *t* test with equal variance. We also analyzed the effect of treatment (conventional, organic) on the number of obtained isolates as well as on the number of species they represent, based on isolates of the “core” community alone (*fide* [18])—including representatives of the genera *Alternaria* Nees; *Aureobasidium* Viala & G. Boyer; *Cladosporium* Link; *Filobasidium* L.S. Olive; *Naganishia* Goto; *Papiliotrema* J.P. Samp., M. Weiss & R. Bauer; *Rhodotorula* F.C. Harrison; *Sampaiozyma* Q.M. Wang, F.Y. Bai, M. Groenew. & Boekhout; *Sporobolomyces* Kluyver & C.B. Niel; *Symmetrospora* Q.M. Wang, F.Y. Bai, M. Groenew. & Boekhout; and *Vishniacozyma* Xin Zhan Liu, F.Y. Bai, M. Groenew. & Boekhout. All statistical analyses were done in Excel 2016 (Microsoft, Redmond, WA, USA) using the XLMiner Analysis ToolPak App (Frontline Systems Inc., Incline Village, NV, USA) downloaded from the Microsoft Office store.

### 2.4. Phylogenetic Analyses

In many genera, a simple BLAST query will not be sufficient for accurate species identification. This is because no single sequence similarity threshold will be able to distinguish intraspecific from interspecific variability in all fungi [19], especially yeasts [20]. For most genera in the core community, we constructed a dataset of ex-type sequences of most similar taxa downloaded from GenBank and supplemented with our newly generated sequences (strains and GenBank accession numbers in Supplementary Table S1). Alignments were constructed using MUSCLE v. 3.7 [21], available on the CIPRES Science Gateway v. 3.3 [22]. After alignment of the ITS datasets, partial SSU and partial LSU were removed by looking for the motifs 5′-ATCATTA-3′ (3′ end of SSU) and 5′-TGACCT-3′ (5′ start of LSU) and deleting downstream and upstream sequence data, respectively [23]. Phylogenetic relationships were inferred by maximum likelihood (ML). We used the command-line version of IQ-TREE [24,25]. Appropriate models of nucleotide substitution were selected according to the corrected Akaike Information Criterion (AICc) through ModelFinder [26] (Table 1). Ultrafast bootstrapping was done with 1000 replicates [27]. Final trees with ML bootstrap support values (BS) were visualized in FigTree (http://tree.bio.ed.ac.uk/software/figtree/, accessed on 7 April 2021) and edited in Adobe Illustrator 24.1.1 (San Jose, CA, USA).

## 3. Results

### 3.1. Culture-Based Community from Romaine Lettuce

A total of 331 fungal isolates were obtained from 42 romaine lettuce samples; 154 isolates were obtained from 14 organic lettuce samples, 147 from 25 conventional samples and 30 from three hydroponic samples. ITS barcode sequences were generated for 249 of these isolates, representing 59 species in 28 genera (Appendix A, Table A1). At the level of phylum, the majority of isolates were Basidiomycota (63.5%), followed by Ascomycota (36.1%) and Mucoromycota (0.4%, a single isolate). The subphylum that was most represented among lettuce isolates was Pezizomycotina (35.0%), followed by Agaricomycotina (32.5%) and Pucciniomycotina (30.1%). The other subphyla in our dataset were represented by one to three isolates: Mucoromycotina, Saccharomycotina, Ustilaginomycotina. In terms of growth, yeast-like fungi were most represented with 170 isolates (68%), compared to 79 isolates of filamentous fungi (32%). Filamentous fungi were distributed in Pezizomycotina (77 isolates), Agaricomycotina (1) and Mucoromycotina (1). The yeast-like fungi were distributed in Agaricomycotina (80 isolates), Pucciniomycotina (75), Pezizomycotina (10), Saccharomycotina (3) and Ustilaginomycotina (2).

**Figure 1:**
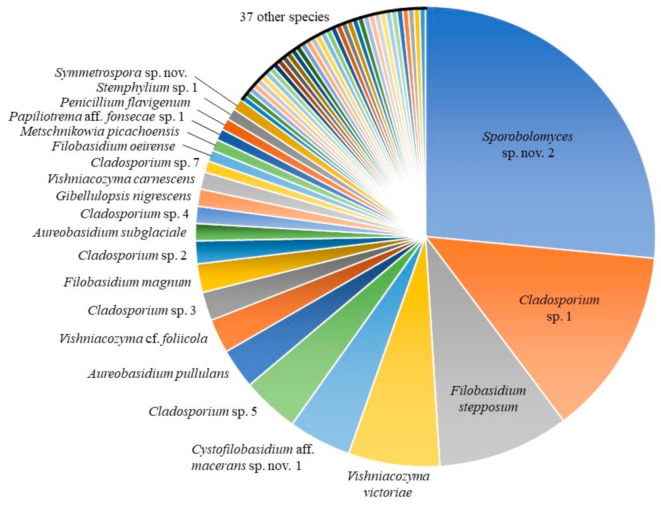
Overview of the 59 species obtained from all lettuce samples analyzed in this study, by number of isolates. Only species with 2 or more isolates are labeled.

**Figure 2:**
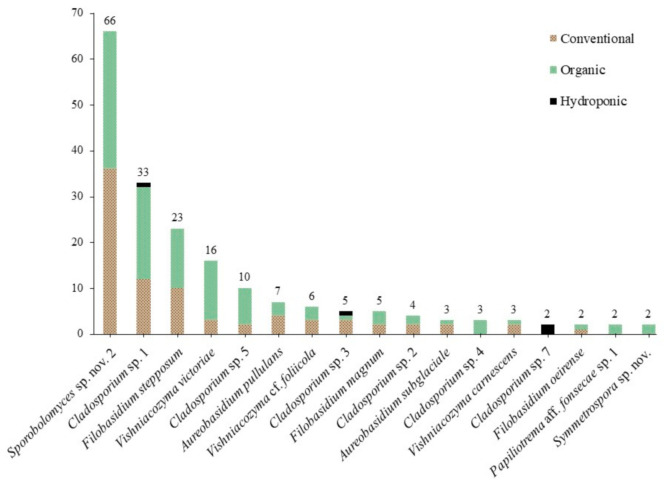
Number of isolates by treatment (conventional, organic, hydroponic) for “core” community species with 2 or more isolates.

The most abundant taxa in our culture-based community belonged to the genus *Sporobolomyces* (26.9%), followed by *Cladosporium* (23.7%), *Filobasidium* (13.3%), *Vishniacozyma* (10.8%), *Cystofilobasidium* Oberw. & Bandoni (4.8%) and *Aureobasidium* (4.0%) (Table 2). Fifty-nine species were identified using the ITS barcode region (Figure 1). The highest number of isolates obtained for a single species was 66: an undescribed species of the red-yeast genus *Sporobolomyces* (Pucciniomycotina, Microbotryomycetes), with 36 isolates from 16 conventional lettuce samples and 30 from 11 organic samples (Figure 2). We obtained isolates of this species from 27 of 42 lettuce samples (64.3%). Five other species were abundant, with 10 or more isolates: 33 isolates of *Cladosporium* sp. 1 and 10 isolates of *Cladosporium* sp. 5 (Pezizomycotina, Dothideomycetes); 23 isolates of *Filobasidium stepposum* (Golubev & J.P. Samp.) Xin Zhan Liu, F.Y. Bai, M. Groenew. & Boekhout; 16 isolates of *Vishniacozyma victoriae* (M.J. Montes, Belloch, Galiana, M.D. García, C. Andrés, S. Ferrer, Torr.-Rodr. & J. Guinea) Xin Zhan Liu, F.Y. Bai, M. Groenew. & Boekhout; and 11 isolates of *Cystofilobasidium* aff. *macerans* J.P. Samp. sp. nov. 1 (Agaricomycotina, Tremellomycetes). Between two and seven isolates were obtained for sixteen species and 37 species were found only once (one isolate each).

### 3.2. Statistical Analyses

**Figure 3:**
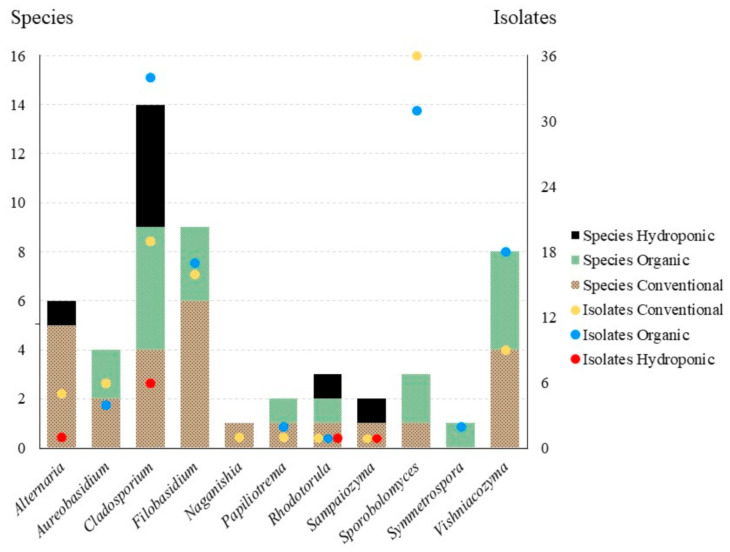
Number of species (bars) and isolates (circles) by treatment (conventional, organic, hydroponic) for “core” community genera.

We obtained a total of 110 isolates representing 34 species from conventional lettuce samples, 128 isolates and 33 species from organic lettuce samples and eleven isolates and ten species from the hydroponic samples. Effect of treatment was significant among all samples (three treatments, F(2,174) = 3.9067, *p* = 0.0219), but it was not significant when only isolates from conventional and organic lettuce samples were compared (to treatments, *t*(116) = 0.3266, *p* = 0.7445). Of the 11 genera in the core community, 10 were obtained from conventional lettuce samples (95 isolates, 26 species), eight from organic samples (109 isolates, 19 species) and four from hydroponic samples (nine isolates, eight species) (Figure 3). For the core community, the effect of treatment on both the number of isolates (2 treatments, *t*(20) = 0.2473, *p* = 0.8072) and the number of species (*t*(20) = 0.8051, *p* = 0.4302) was not significant.

### 3.3. Taxonomy

#### 3.3.1. *Aureobasidium bupleuri* (Bills) Haelew. & Aime, comb. nov.

MycoBank MB835676.

Basionym: *Kabatiella bupleuri* Bills, Mycologia 104 (4): 966 (2012).

**Figure 4:**
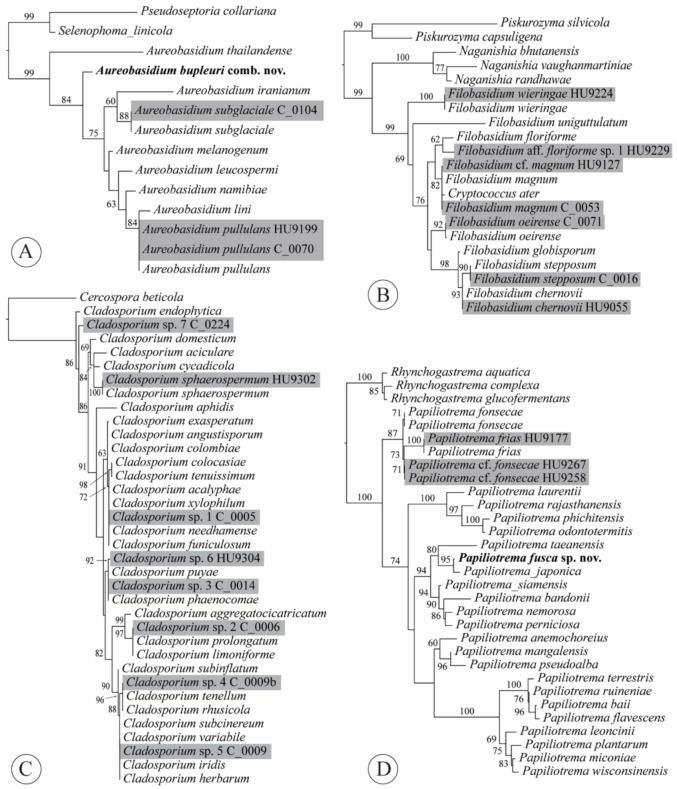
Phylogenetic reconstructions based on ITS sequences for the following genera: (**A**), *Aureobasidium*; (**B**), *Filobasidium*; (**C**), *Cladosporium*; and (**D**), *Papiliotrema*. Topologies are the result of ML inference performed with IQ-TREE. For each node, the ML bootstrap (if ≥60) is presented above/below the branch leading to that node. Gray shading added for strains obtained from romaine lettuce samples during this study.

Notes: The combination was based on phylogenetic evidence. Taxonomy of the black yeasts has been traditionally confusing, with characters that are usually important in the classification of asexual morphs being phylogenetically uninformative in Dothideales. In our ITS tree, the ex-type sequence of *K. bupleuri* (GenBank acc. no. NR_121524 [28]) was nested within *Aureobasidium* (Figure 4A). The same result was found based on an ITS phylogeny, as well as an *rpb2* phylogeny that both included ex-type sequences of *K. bupleuri* [29]. Both analyses also included the type species of *Kabatiella*, *K. microsticta* Bubák, which was retrieved within *Aureobasidium* with high support.

#### 3.3.2. *Curvibasidium nothofagi* (C. Ramírez & A.E. González) Haelew. & Aime, comb. nov.

MycoBank MB835657.

Basionym: *Rhodotorula nothofagi* C. Ramírez and A.E. González, Mycopathologia 91 (3): 171 (1985).

Notes: The combination was made based on phylogenetic evidence. Kurtzman et al. [30] presented a phylogeny with sequences of the large subunit (LSU) ribosomal RNA gene that retrieved *R. nothofagi* in the *Curvibasidium* Samp. & Golubev clade [31] together with *Curvibasidium cygneicollum* J.P. Samp. and *C. pallidicorallinum* Golubev, Fell & N.W. Golubev with high support. In addition, blasting the ex-type ITS sequence (GenBank acc. no. AB038096, CBS:8166, JCM:9304; T. Nagahama et al. unpubl.) against other sequences from type materials, resulted in *C. cygneicollum* (NR_111077, CBS:4551 [32]) and *C. pallidicorallinum* (KY102982, CBS:9091 [20]) with the highest percentages of identity (98.86% and 99.48%, respectively).

#### 3.3.3. *Filobasidium magnum* (Lodder & Kreger-van Rij) Xin Zhan Liu, F.Y. Bai, M. Groenew. & Boekhout, in Liu et al., Stud. Mycol. 81: 118 (2015)

Basionym: *Cryptococcus laurentii* var. *magnus* Lodder & Kreger-van Rij, in Lodder and Kreger-van Rij, The yeasts: a taxonomic study [Edn 1] (Amsterdam): 670 (1952).

Synonyms: *Cryptococcus ater* (Castell. ex W.B. Cooke) Phaff & Fell, in Lodder, The yeasts: a taxonomic study, 2nd edn (Amsterdam): 1120 (1970), syn. nov.

*Cryptococcus laurentii* f. *ater* Castell. ex W.B. Cooke, Mycopath. Mycol. Appl. 30: 351 (1966), syn. nov.

*Cryptococcus magnus* (Lodder & Kreger-van Rij) Baptist and Kurtzman, Mycologia 68(6): 1200 (1977) [1976].

Notes: *Cryptococcus laurentii* (Kuff.) C.E. Skinner was combined as *Papiliotrema laurentii* (Kuff.) Xin Zhan Liu, F.Y. Bai, M. Groenew. & Boekhout by Liu et al. [33] but *C. ater* [= *C. laurentii* f. *ater*] represented another species. The new synonymy was proposed based on sequence data and phylogenetic evidence. The ex-type ITS sequences of *C. ater* (GenBank acc. no. NR_144813; P. Gujjari and J. Zhou unpubl.) and *F. magnum* (NR_130655 [34]) were 98.91% identical, indicating a single species. Fell et al. [34] presented two phylogenies, one based on ITS, the other based on LSU, that both found *C. ater* and *C. magnus* to be a single species, which was confirmed by our analysis (Figure 4B). However, the synonymy was not formalized until now. Or it was incorrectly implemented; *C. ater* and *C. laurentii* f. *ater* were listed as synonyms of *P. laurentii* [35].

#### 3.3.4. *Papiliotrema fusca* J.P. Samp., J. Inácio, Fonseca and Fell ex Haelew., sp. nov.

MycoBank MB836093

For description see: Int. J. Syst. Evol. Microbiol. 54 (3): 989 (2004).

Holotype: PYCC 5690, permanently preserved in a metabolically inactive state at the Portuguese Yeast Culture Collection. Ex-type culture: CBS 9648.

Synonyms: *Auriculibuller fuscus* J.P. Samp., J. Inácio, Fonseca & Fell, Int. J. Syst. Evol. Microbiol. 54 (3): 989 (2004), *nom. inval.*, Art. 40.6 (Shenzhen).

*Papiliotrema fusca* (J.P. Samp., J. Inácio, Fonseca & Fell) Xin Zhan Liu, F.Y. Bai, M. Groenew. & Boekhout, Stud. Mycol. 81: 126 (2015), *nom. inval.*, Art. 40.6 (Shenzhen).

## 4. Discussion

Previous culture-based studies have found that the phylloplane of crops is dominated by species of *Acremonium* Link, *Alternaria*, *Aureobasidium*, *Cladosporium* (Ascomycota, all filamentous), *Cryptococcus* Vuill., *Rhodotorula* and *Sporobolomyces* (Basidiomycota, all yeast-like) [18,36,37,38,39]. We note that according to pre-2015 generic concepts some of these genera were polyphyletic. Many of these taxonomic problems are now resolved based on multi-locus phylogenies and new genera were introduced to accommodate orphaned species, including *Filobasidium*, *Naganishia*, *Papiliotrema*, *Vishniacozyma* (Tremellomycetes), *Sampaiozyma* (Microbotryomycetes) and *Symmetrospora* (Cystobasidiomycetes) [31,33]. Under these modern concepts, our results are consistent with previous work; the 213 isolates of *Alternaria*, *Aureobasidium*, *Cladosporium*, “*Cryptococcus*” [as *Filobasidium*, *Naganishia*, *Papiliotrema* and *Vishniacozyma*], *Rhodotorula* [in part as *Sampaiozyma*] and *Sporobolomyces* [in part as *Symmetrospora*] represent 85.5% of total isolates and, as a result, can be considered the core community [*fide* 18] (Figure 3). Of these isolates, the yeast-like taxa dominate (64.8% of the core community, while only 55.4% of the total culture-based dataset). We will discuss these genera below.

### 4.1. Alternaria

We obtained six isolates of *Alternaria* (Dothideomycetes, Pleosporales, Pleosporaceae), each representing a different species—five isolates were from conventional lettuce samples, one was from a hydroponic sample. *Alternaria* is a large genus of filamentous fungi with about 366 accepted species [40]. Species are commonly found on a wide range of foods, mostly under the names *A. alternariae* (Cooke) Woudenb. & Crous and *A. tenuis* Nees [syn. of *Alternaria alternata* (Fr.) Keissl.]. Several species are major pathogens of fresh fruit, eggplants, peppers and fresh vegetables [1,41].

The utility of ITS as a barcode for separating species in this genus was questioned earlier [42,43]. We have encountered this issue during our work with *Alternaria* isolates from romaine lettuce. For example, the ITS sequence of isolate HU9315 from a hydroponic lettuce sample was 100% similar to both ex-type sequences of *A. angustiovoidea* E.G. Simmons and *A. destruens* E.G. Simmons. We went one step further and compared ex-type sequences for the glyceraldehyde 3-phosphate dehydrogenase (*gpd*) gene to find that *A. angustiovoidea* (GenBank acc. no. JQ646315 [44]) and *A. destruens* (AY278812 [45]) can be separated from each other with that marker, with 1.04% nt differences between the two species. Since our study was based on ITS alone, we did not assign names to our isolates. Instead, we referred to them as *Alternaria* spp. 1–6 (Appendix A, Table A1). They are most close to *A. angustiovoidea*; *A. arbusti* E.G. Simmons; *A. californica* E.G. Simmons & S.T. Koike; *A. conjuncta* E.G. Simmons; *A. dactylidicola* Thambug., Camporesi & K.D. Hyde; *A. destruens*; *A. eureka* E.G. Simmons; *A. oregonensis* E.G. Simmons; *A. prasonis* E.G. Simmons; and *A. ventricose* R.G. Roberts. To our knowledge, none of these have been reported on lettuce or any member of Asteraceae before. We note that artificial transmission of *Alternaria* to atypical hosts has been reported. For example, *A. dauci* (J.G. Kühn) J.W. Groves & Skolko, causing leaf blight on carrot (*Daucus carota* L., Apiaceae), has been shown to infect non-Apiaceae hosts including lettuce [46,47,48].

Of the potential species in our dataset, only *A. arbusti* has a wide host range, including species in the genera *Hordeum* L., *Phleum* L. and *Triticum* L. (Poaceae); *Prunus* L. and *Pyrus* L. (Rosaceae); *Sesbania* Scop. (Fabaceae); and *Solanum* L. (Solanaceae). However, it had not been reported from lettuce before [49,50,51,52]. These findings highlight the need for additional taxonomic work within *Alternaria* and research on the production of toxins and their host-selectiveness for each species, from a public health standpoint.

### 4.2. Aureobasidium

We obtained ten isolates of *Aureobasidium* (Dothideomycetes, Dothideales, Saccotheciaceae), six isolates from six conventional lettuce samples and four isolates from three organic samples. Seven isolates represented *A. pullulans* (de Bary & Löwenthal) G. Arnaud, based on 100% similarity matches with the ITS sequence generated for the neotype [53]. *Aureobasidium* currently comprises 26 species, of which only *A. pullulans* is important in the food industry [1,40,54]. The black yeast-like *A. pullulans* is a ubiquitous saprobe, especially found on cold-stored and frozen foods as well as fresh produce. It was isolated from a wide range of vegetables but not Cucurbitaceae [55]: green beans, mustard greens, turnip greens, asparagus, broccoli, cabbage, chinese cabbage, cauliflower, kale, lettuce, okra and radish. *Aureobasidium pullulans* has applications in biological control [56], e.g., against postharvest *Penicillium* rot in apple and citrus [57,58] and fire blight caused by *Erwinia amylovora* (Burril) Winslowfire et al. in pome fruit trees [59].

The other species in our dataset, represented by three isolates, was *A. subglaciale* (Zalar, de Hoog & Gunde-Cim.) Zalar, de Hoog & Gunde-Cim. (Figure 4A, Appendix A, Table A1). The type of this species was isolated from subglacial ice from sea water in Norway. Given its psychrotolerant nature and exclusive presence in the Arctic [60], it is quite surprising to find three isolates of this species from three of our North American romaine lettuce samples—two from conventionally grown lettuce samples and one from organic lettuce. This may indicate that some treatments, such as refrigeration, may favor growth of microbes that are adapted for different climatic conditions and would otherwise not be able to compete on the phylloplane.

### 4.3. Cladosporium

Of 77 isolates of Dothideomycetes, 59 represented species of *Cladosporium* (Capnodiales, Cladosporiaceae). Those isolates were obtained from 28 of 42 lettuce samples (66.7%). Nineteen isolates were obtained from twelve conventional lettuce samples, 34 isolates from thirteen organic samples and six isolates from three hydroponic ones. Species of *Cladosporium* are among the most abundant fungi in outdoor and indoor air [61,62,63,64,65]; it is no surprise that the second-highest number of isolates from lettuce samples were *Cladosporium*. This genus currently includes 237 accepted species isolated from a range of substrates including plant material, soil, the air, food, building materials and clinical samples [40,65,66,67]. Species accepted in *Cladosporium* occur as pathogens on fresh fruit and vegetables, causing spoilage of strawberries, tomatoes and refrigerated cheese and meat. In other instances, they are merely colonizers [1,66].

Phylogenetic studies have shown that many morphologically circumscribed species comprise several cryptic species. Based on morphology, most species of *Cladosporium* can be assigned to one of three major species complexes—*C. cladosporioides* (Fresen.) G.A. de Vries, *C. herbarum* (Pers.) Link and *C. sphaerospermum* Penz. [65,68,69,70]. Using molecular data has improved understanding of species limits; the most informative phylogenetic markers are the actin gene (*actA*) and translation elongation factor 1-α (*tef1*) [66]. The ITS alone only provides accuracy to species complex-level; ITS sequences are usually identical for species belonging to the same species complex [71].

Five clusters of ITS sequences from our isolates were identical to ex-type sequences of 3–18 different species within either the *C. cladosporioides* complex or the *C. herbarum* complex (Figure 4C, Appendix A, Table A1), supporting previous findings [71]. *Cladosporium* sp. 1 is part of the *C. cladosporioides* complex, whereas *Cladosporium* spp. 2–5 all belong in the *C. herbarum* complex. Two isolates of *Cladosporium* sp. 7 from a hydroponic lettuce sample shared 99.11% identity with the ex-type sequence of *C. endophyticum* Tibpromma & K.D. Hyde, an endophytic species isolated from leaves of *Pandanus* sp. in Thailand. Finally, the ITS sequence of isolate HU9302 from a hydroponic lettuce sample shared 100% identity with the ex-neotype sequence of *C. sphaerospermum*, representing the only species of *Cladosporium* that we could identify with certainty. This species has been reported from a wide range of food, but is apparently less common than *C. cladosporioides* [1].

### 4.4. Filobasidium

A total of 33 isolates representing six species of the yeast genus *Filobasidium* (Tremellomycetes, Filobasidiales, Filobasidiaceae) were obtained from 20 lettuce samples. Sixteen isolates were obtained from eleven conventional lettuce samples, 17 isolates from nine organic samples. *Filobasidium* currently contains 11 species isolates from leaves and florets of various plants, weathered inflorescence scapes of *Yucca* L., apples, grapes (including grape juice and must), *Euploea* Fabricius butterflies, sake moto, cider, breweries/wineries, cheeses, raw milk and forest soil [30,33,72,73,74,75,76]. In addition, *F. globosporum* Bandoni & Oberw. and *F.* sp. (as “*elegans*”) have been identified in the intestines of humans and mice [77,78], whereas *F. uniguttulatum* Kwon-Chun isolates have been obtained from a patient with meningitis and from cloaca and fresh dropping samples of feral pigeons [79,80].

Li et al. [76] primarily used LSU for species delimitation phylogenetic analyses, but these authors also discussed number of nucleotide mismatches among ITS sequences in their assessments of species limits. A ≥ 3% mismatch in the ITS for a certain isolate with an ex-type was considered as an indication of that isolate representing a separate species. Following the same strategy, our isolates represent *F. chernovii* (Á. Fonseca, Scorzetti & Fell) Xin Zhan Liu, F.Y. Bai, M. Groenew. & Boekhout (one isolate); *F. magnum* (Lodder & Kreger-van Rij) Xin Zhan Liu, F.Y. Bai, M. Groenew. & Boekhout (five isolates); *F. oeirense* (Á. Fonseca, Scorzetti & Fell) Xin Zhan Liu, F.Y. Bai, M. Groenew. & Boekhout (two isolates); *F. stepposum* (Golubev & J.P. Samp.) Xin Zhan Liu, F.Y. Bai, M. Groenew. & Boekhout (23 isolates) and *F. wieringae* (Á. Fonseca, Scorzetti & Fell) Xin Zhan Liu, F.Y. Bai, M. Groenew. & Boekhout (one isolate) (Appendix A, Table A1). In addition, we isolated a single isolate (HU9229) of *F.* aff. *floriforme* L.S. Olive sp. 1. Compared to the ex-type sequence of *F. floriforme*, there were four mismatches and one gap (99.06% identity). Even though support was low, the evolutionary distance between the two isolates in our ML tree of *Filobasidium* ITS sequences (Figure 4B) showed that they are likely not the same species. All our isolates are the first ones from romaine lettuce. As most species appear to be non-pathogenic to humans and animals [30], the isolates we obtained may be merely environmental contaminants of the lettuce surface.

### 4.5. Naganishia

**Figure 5:**
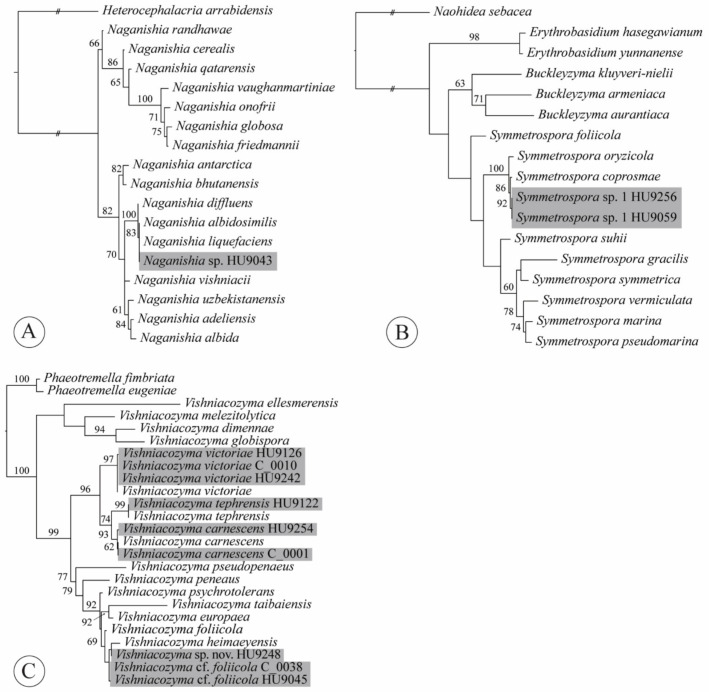
Phylogenetic reconstructions based on ITS sequences for the following genera: (**A**), *Naganishia*; (**B**), *Symmetrospora*; and (**C**), *Vishniacozyma*. Topologies are the result of ML inference performed with IQ-TREE. For each node, the ML bootstrap (if ≥60) is presented above/below the branch leading to that node. Gray shading added for strains obtained from romaine lettuce samples during this study.

We isolated a single strain of *Naganishia* sp. (Tremellomycetes, Filobasidiales, Filobasidiaceae) from a conventional lettuce sample (Figure 5A, Appendix A, Table A1). The genus *Naganishia* was described to accommodate the yeast *N. globosa* Goto [81]. Liu et al. [33], based on an LSU phylogeny, synonymized 14 species that previously belonged to *Cryptococcus* (*albidus* clade (*sensu* [82])). Currently, 18 species of *Naganishia* are accepted [76,83,84,85]. Species in this genus have been isolated from air, water, soil, cold environments (glaciers, snow, cryoconite), plant surfaces, cheese, fermented cereals and a purification tank for polluted water [83,86]. Three species are reported from clinical samples: *Naganishia diffluens* (Zach) Xin Zhan Liu, F.Y. Bai, M. Groenew. & Boekhout; *N. globosa* [syn. *Cryptococcus saitoi* Á. Fonseca, Scorzetti & Fell] and *N. liquefaciens* (Saito & M. Ota) Xin Zhan Liu, F.Y. Bai, M. Groenew. & Boekhout [30]. Distinction among *N. albidosimilis* (Vishniac & Kurtzman) Xin Zhan Liu, F.Y. Bai, M. Groenew. & Boekhout, *N. diffluens* and *N. liquefaciens* can be best achieved using LSU (D1/D2 loop region); the three species differ by 0–1 nt substitutions in the ITS. The sequence of our isolate HU9043 is 100% identical to both *N. albidosimilis* and *N. liquefaciens*. According to Kurtzman et al. [30], identifications of *N. albidosimilis*, *N. diffluens* and *N. liquefaciens* based solely on ITS sequences are best considered tentative. This is also visible from our ITS tree (Figure 5A), in which isolate HU9043 and the ex-type sequences of both *N. albidosimilis* and *N. liquefaciens* are placed together with high support, sister to *N. diffluens*. Neither of these species have thus far been found on leaves of lettuce.

### 4.6. Papiliotrema

We obtained three isolates of *Papiliotrema* (Tremellomycetes, Tremellales, Rhynchogastremaceae), two isolates of *P.* aff. *fonsecae* (V. de García, Zalar, Brizzio, Gunde-Cim. & Van Broock) Yurkov sp. 1 and one isolate of *P. frias* V. de García, Zalar, Brizzio, Gunde-Cim. & van Broock ex Yurkov (Appendix A, Table A1). Yeasts in the genus *Papiliotrema* have been isolated from inflorescences and leaf tissues of plants, trees, termite guts, subglacial ice and meltwater from glaciers, water samples and soil [87,88,89,90,91,92,93]. Interestingly, *P. siamensis* Suruss. & Limtong was found as both an epiphyte and an endophyte [91]. It appears that both ITS and LSU can be used for species delimitation in this genus; Pagani et al. [93] presented comparable nucleotide differences among species in the ITS and LSU regions. The phylogenetic reconstruction of an ITS dataset with our isolates and ex-type collections resolved all species described to date (Figure 4D). Isolate HU9177 obtained from a conventional lettuce sample was conspecific with *P. frias*. Isolates HU9258 and HU9267 (Figure 4D) are most similar with the ex-type sequence of *P. fonsecae* (99.76%, 1 nt different). However, they were retrieved as a sister clade to *P. frias*, but with considerable evolutionary distance. The distance between *P. fonsecae* isolates (EXF-4087 and ZM13F84) and our lettuce isolates was negligible, but it is likely that our isolates represent an undescribed species, which we here refer to as *P.* aff. *fonsecae* sp. 1. We note that we included a non-type sequence of *P. fonsecae* in this tree, because the ex-type sequence for *P. fonsecae* was very short and was retrieved in different positions without support in preliminary trees.

### 4.7. Rhodotorula

*Rhodotorula* (Microbotryomycetes, Sporidiobolales, Sporidiobolaceae) is one of two genera (*Sporobolomyces* being the other one) in which asexual red-pigmented yeasts were artificially placed based on morphology and physiology [94,95,96]. Both these genera are polyphyletic in their traditional sense [34,97,98] and representatives of *Rhodotorula* s.l. are retrieved in 17 different clades in Cystobasidiomycetes, Microbotryomycetes and Ustilaginomycetes [97,99]. Consequently, 27 species of *Rhodotorula* were recombined in 15 genera, leaving 19 accepted species in *Rhodotorula* emend. [31]. In our assessment of the genus, we found that *R. nothofagi* should be combined in *Curvibasidium* based on both ITS and LSU sequence data [20,30,32] (see Section 3.3).

**Figure 6:**
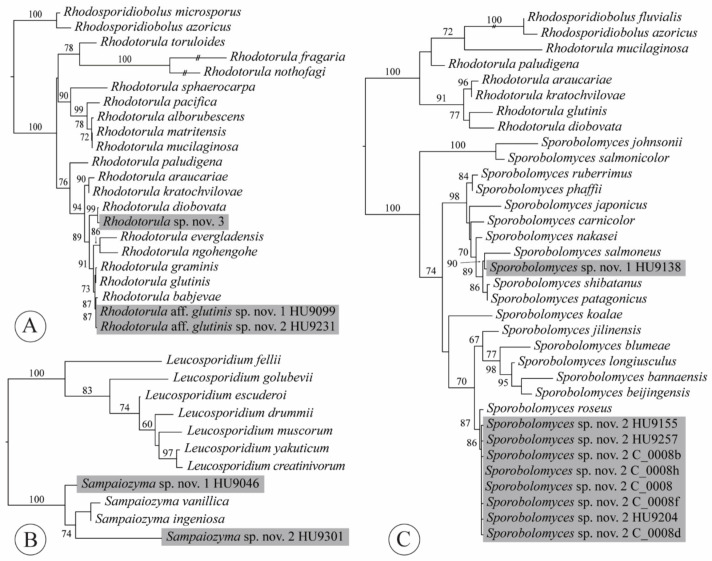
Phylogenetic reconstructions based on ITS sequences for the following genera: (**A**) *Rhodotorula*; (**B**) *Sampaiozyma*; and (**C**) *Sporobolomyces*. Topologies are the result of ML inference performed with IQ-TREE. For each node, the ML bootstrap (if ≥60) is presented above/below the branch leading to that node. Gray shading added for strains obtained from romaine lettuce samples during this study.

We obtained three isolates from three lettuce samples, each representative of another, potentially undescribed species (Appendix A, Table A1). Two isolates belonged in the *R. glutinis* sensu stricto group (Figure 6A), which was proposed by Gadanho and Sampaio [100] to accentuate the close phylogenetic relationships among *R. babjevae* (Golubev) Q.M. Wang, F.Y. Bai, M. Groenew. & Boekhout, *R. glutinis* (Fresen.) F.C. Harrison and *R. graminis* Di Menna. According to Kurtzman et al. [30], 1–3 mismatches were found among ITS sequences of these three species. Both isolates HU9099 and HU9231 were most similar to *H. babjevae*, with only two and three mismatches with the ex-type sequence for this species (GenBank acc. no. NR_077096 [101]), respectively. The ITS sequences of isolates HU9099 and HU9231 differed from each other in one nucleotide, which seems sufficient to recognize them as distinct species within the *R. glutinis* sensu stricto clade [*sensu* 30]. Our third isolate (HU9305), from a hydroponic lettuce sample, was most similar to *R. diobovata* (S.Y. Newell & I.L. Hunter) Q.M. Wang, F.Y. Bai, M. Groenew. & Boekhout, with three mismatches—thus, also representing an undescribed species.

*Rhodotorula* is a ubiquitous saprobic genus that is isolated from many different habitats, including in extreme conditions [102]. Gildemacher et al. [103] reported that *Rhodotorula* sp. suppresses other yeast species and increases russet formation on apples. A number of studies have mentioned biocontrol properties of *Rhodotorula* “*glutinis*” although their identifications may be erroneous because this species is rarely isolated compared to other morphologically similar species of red yeast [30]. *Rhodotorula* often occurs on the phylloplane and other types of plant substrates and species have been isolated from lettuce leaves in previous studies [104,105,106,107]. However, we are only aware of one study focusing on romaine lettuce [108]; red romaine lettuce (*Lactuca sativa* cv. “Outredgeous”) was grown in rooting pillows in a plant growth chamber on the International Space Station, harvested, brought back to earth and analyzed to genus-level. Of ten genera isolated from lettuce leaves and roots, *Rhodotorula* was the most common. Urbina and Aime [109] reported the urgency of formally characterizing undescribed species that are constantly consumed. This may be especially important for *Rhodotorula* species, some of which have been reported as emerging (opportunistic) pathogens for both humans and animals [102,110,111,112].

### 4.8. Sampaiozyma

*Sampaiozyma* (Microbotryomycetes *incertae sedis*) was one of the genera introduced by Wang et al. [31] to accommodate red-yeast species previously classified as *Rhodotorula*, isolated from stagnant water and leaf surfaces of pasture plants [113,114]: *S. ingeniosa* (Di Menna) Q.M. Wang, F.Y. Bai, M. Groenew. & Boekhout and *S. vanillica* (J.P. Samp.) Q.M. Wang, F.Y. Bai, M. Groenew. & Boekhout. *Sampaiozyma* is most closely related to *Leucosporidium* Fell, Statzell, I.L. Hunter & Phaff. Wang et al. [99] retrieved the genus—as the “vanillica clade”—as sister to *Leucosporidium* with high support in their ribosomal DNA and seven-locus phylogenies. Liu et al. [115] found high support for the sister relationship of *Leucosporidium* and *Sampaiozyma*. The ex-type ITS sequences of *S. ingeniosa* and *S. vanillica* only differ in three nucleotides. We obtained two isolates of *Sampaiozyma*, one from a conventional lettuce sample, the other from a hydroponic sample. The two isolates each represent an undescribed species (Figure 6B, Appendix A, Table A1). In the ITS sequence, isolate HU9046 had 13 mismatches compared to *S. ingeniosa* and 15 mismatches compared to *S. vanillica*. Isolate HU9301 differed from both species by 37 mismatches. Both lettuce isolates differed from each other by 39 mismatches in the ITS. Formal description of these is pending examination of morphological and physiological characteristics.

### 4.9. Sporobolomyces

Wang et al. [31] revised the red-yeast genus *Sporobolomyces* (Microbotryomycetes, Sporidiobolales, Sporidiobolaceae) because it was polyphyletic in the traditional sense, occurring in most of the yeast-forming classes of Pucciniomycotina, as well as in Agaricomycotina, Ustilaginomycotina and, most recently, in Pezizomycotina [97,99,116]. The authors proposed new combinations of 40 species in 16 different genera. The emended concept of *Sporobolomyces* included sixteen species. Six more species have been described by Lorenzini et al. [117] and Li et al. [76] bringing the number of currently accepted species to 22, although current estimates place the total number of species at 60 for *Sporobolomyces* [109]. The red-yeast genera in the Sporidiobolales produce lipid droplets full of carotenoid pigments, particularly β-carotene and torulene, which contribute to the rich pink to orange-red color of colonies [109,118,119]. These pigments, although poorly understood, seem to protect them against UV radiation and some provide antimicrobial, anticancer and antiaging activity [120,121]. Because of these characteristics, red-pigmented yeasts have gained interest from the pharmaceutical, cosmetics and biotechnology industries [122,123,124].

*Sporobolomyces* species are free-living and worldwide in distribution. They are reported from many different habitats—including freshwater and marine ecosystems, excrements of maize pests, fruit must, soil, buildings and air—although most commonly from the plant phylloplane [109,117,125,126,127,128,129,130,131]. Last [127] suggested an association between colony development and senescence, after observing that numbers of colonies of *Sporobolomyces* start to increase when cereal leaves reach their half-age. The promise of *S. roseus* Kluyver & C.B. Niel as a biocontrol against postharvest diseases of pome fruits has been shown by Janisiewicz et al. [132]. After yeast application to wounded apples, incidence of rots was reduced from 33% to 0% for *Penicillium expansum* Link (blue mold) and from 92% to 4% to *Botrytis cinerea* Pers. (gray mold). Important to note is that, in addition to the leaf age, humidity is also an important factor in the growth of *S. roseus*. Bashi and Fokkema [133] experimentally found that relative humidity of 65% decreased the population of colonies; *Sporobolomyces* can only use nutrients that are present on the leaves at relative humidity of at least 90%.

*Sporobolomyces* was the most abundantly recovered genus in our culture-based community, representing more than a quarter of all isolates (26.9%). Sixty-seven isolates were obtained from 27 of 42 lettuce samples (64.3%). Whereas the number of isolates was the highest of any genus isolated in this study, the number of species was only two. Both species appeared to be undescribed (Figure 6C, Appendix A, Table A1). *Sporobolomyces* sp. nov. 1 was represented by a single isolate (HU9138), obtained from an organic lettuce sample. It shares 98.82% identity with *S. patagonicus* Libkind, Van Broock & J.P. Samp. isolated from subsurface water in Argentina. *Sporobolomyces* sp. nov. 2 was detected on eleven organic lettuce samples (30 isolates) and sixteen conventional samples (36 isolates). Through a BLAST search in NCBI GenBank (100% identity hits), we found that this same yeast has a broad geographic distribution and is found from different habitats: on a wilting leaf of *Parthenocissus* sp. (Vitales, Vitaceae) in China [134], *Prunus avium* L. cherries (Rosales, Roaceae) in Spain [135], *Vitis vinifera* L. grapes (Vitales, Vitaceae) in Washington [136], glacier rocks in Antarctica [137], *Quercus faginea* Lam. leaves (Fagales, Fagaceae) in Portugal [20], *Antirrhinum* L. flowers (Lamiales, Plantaginaceae) in California, *Septoria blasdalei*-diseased leaves of *Ceanothus arboreus* Greene (Rosales, Rhamnaceae) in California [109] and guava juice in Egypt (Z.S.M. Soliman unpubl.).

### 4.10. Symmetrospora

From two organic lettuce samples, we obtained two isolates of an undescribed species of *Symmetrospora* (Cystobasidiomycetes *incertae sedis*, Symmetrosporaceae). *Symmetrospora* was introduced by Wang et al. [31] for species that were previously placed in the polyphyletic red-yeast genera *Rhodotorula* and *Sporobolomyces* of the “*gracilis*/*marina* clade” [34,97,99,138,139]. Haelewaters et al. [140] and Li et al. [76] recently proposed four new species and a new combination of *Symmetrospora*, bringing the total to 11 recognized species. *Symmetrospora* is known in North and South America, Europe, Asia and Australia and species in the genus have been isolated from a wide diversity of habitats—leaf surfaces, air, marine water, beetle guts and a sea sponge [76,125,128,140,141,142,143,144,145]. Our two isolates were previously reported as *S.* cf. *coprosma* [140]. These isolates, HU9059 and HU9256, are most similar (99.48% identity) to the ex-type sequence of *S. coprosmae* (Hamam. & Nakase) Q.M. Wang, F.Y. Bai, M. Groenew. & Boekhout (GenBank acc. no. NR_073317 [32]) but seem to represent a distinct, undescribed species based on the phylogenetic reconstruction of an ITS dataset (Figure 5B). Formal description of this species is pending the characterization of morphology and physiology [140,146].

### 4.11. Vishniacozyma

In our dataset, we obtained 27 isolates of *Vishniacozyma* (Tremellomycetes, Tremellales, Bulleribasidiaceae) from 18 lettuce samples, of which nine isolates were obtained from eight conventional lettuce samples and 18 from ten organic ones. Liu et al. [33], in their phylogenetic classification of Tremellomycetes, introduced *Vishniacozyma* to accommodate eleven species previously known as members of *Bullera* Derx and *Cryptococcus*. Currently, the genus *Vishniacozyma* contains sixteen species [33,76,147,148]. Species in the genus are isolated from different sources—including flowers and plant leaves, sediments and surface sediments of a retreating glacier and soil. According to Li et al. [76], 9 or more nucleotide differences (1.9% mismatches) among ITS sequences can be considered as indication for different taxa. A total of five species were represented in our dataset (Figure 5C, Appendix A, Table A1): *Vishniacozyma carnescens* (Verona & Luchetti) Xin Zhan Liu, F.Y. Bai, M. Groenew. & Boekhout (three isolates); *V.* cf. *foliicola* Q.M. Wang & F.Y. Bai ex Yurkov (6 isolates); *V.* aff. *heimaeyensis* Vishniac ex Xin Zhan Liu, F.Y. Bai, M. Groenew. & Boekhout sp. nov. (one isolate); *V. tephrensis* Vishniac ex Xin Zhan Liu, F.Y. Bai, M. Groenew. & Boekhout (one isolate); and *V. victoriae* (M.J. Montes, Belloch, Galiana, M.D. García, C. Andrés, S. Ferrer, Torr.-Rodr. & J. Guinea) Xin Zhan Liu, F.Y. Bai, M. Groenew. & Boekhout (16 isolates). Isolate HU9248 had seven mismatches with *V. heimaeyensis* in its ITS, which indicated that it represented a different species, which was confirmed by the evolutionary distance among both isolates revealed from the ITS phylogenetic analysis (Figure 5C). Our ITS sequences of C_0038 and HU9045 were 98.67–98.86 identical to the ex-type sequence of *V. foliicola*, marking 6–7 mismatches. The placement of these isolates in our ITS tree had no support, which is why we marked them as *V.* cf. *foliicola*, pending detailed study of morphological and physiological characteristics.

## 5. Conclusions

Lettuce leaves are nearly always consumed raw rather than cooked. As a result, the microbes that colonize the lettuce phylloplane are mostly ingested by the consumer. Despite this, our knowledge about natural fungal species associated with the phylloplane of romaine lettuce is still very limited, yet this knowledge is necessary for understanding how this naturally occurring flora interacts with introduced human pathogens on the phylloplane. Thus, characterizing these microbial communities is crucial from a public health point of view. In addition, the lettuce industry in the US is worth 6 billion USD annually [5], which adds a not to be underestimated economic factor to the balance. Both factors have led to an interest in the native mycota on the romaine lettuce phylloplane. Here, we characterized the culturable fungal microorganisms on the leaf surface of romaine lettuce using a culture-dependent study combined with molecular identification based on the fungal barcode [149]. We obtained 331 isolates from 42 romaine lettuce samples and generated barcode sequences (ITS) for 249 isolates, representing 59 species. Of these, 12 are undescribed species, all yeasts. This is no surprise; Kachalkin et al. [85] reported that “it becomes challenging for researchers to maintain an overview of the ever-growing diversity of undescribed yeasts”, because countless biodiversity studies report undescribed yeast species. Yeasts are isolated from all around the world and from a plethora of habitats and substrates [150]. Especially the phylloplane represent a popular substrate for (asymptomatic, presumably, saprobic) yeasts. Indeed, plant leaf surfaces represent one of the largest terrestrial microbial habitats; the estimated total surface area available for colonization is 200–640 million km^2^ [151,152]. Compared to filamentous fungi, yeasts appear to colonize plant leaf surfaces more actively [86,153].

The core fungal community on romaine lettuce includes 38 species in 11 genera (Alternaria, Aureobasidium, Cladosporium, Filobasidium, Naganishia, Papiliotrema, Rhodotorula, Sampaiozyma, Sporobolomyces, Symmetrospora and Vishniacozyma). Within this core community, the yeast-like taxa dominate (with 64.8% of isolates). Interestingly, management treatment (conventional vs. organic) has no effect on the number of fungal isolates (t(20) = 0.2473, p = 0.8072), nor on the number of species (t(20) = 0.8051, p = 0.4302). However, we emphasize that these results may be biased due to the nature of this study; we may not have cultured every species that was effectively present on lettuce leaves (due to selective media, slow-growing species, etc.). Treatments potentially driving changes in fungal communities are best studied using a high-throughput sequencing approach (D. Haelewaters and M.C. Aime, in preparation).

Based on over a decade of fieldwork in understudied habitats and regions, Urbina and Aime [109] estimated that a meager 16% of red yeast species in Sporidiobolales have been described to date. For one species of Sporidiobolales, an undescribed species of *Sporobolomyces*, we recovered 66 isolates from 27 romaine lettuce samples. This species represented 26.5% of our total isolates and 31.0% of our “core” culture-based community. An earlier study also reported *Sporobolomyces* to be a dominant portion of the lettuce phylloplane, with 35.5% to 63.0% representation of the total community depending on the cultivar [18]. It is important to formally characterize this undescribed species, which is presumably often consumed with romaine lettuce. Provided its dominance, this species should be considered a candidate for experimental work assessing interactions with human pathogens. To mitigate future outbreaks, it is crucial to understand the ability of *E. coli* to enter and persist in these phylloplane fungal communities as part of an outbreak control strategy.

## Figures and Tables

**Table 1:** Features of sequence datasets and results of maximum likelihood (ML) inference by genus. Presented are the number of included isolates, number of total characters, number and percentage of parsimony-informative characters, the model of nucleotide substitution according to the corrected Akaike Information Criterion and the -lnL score of the best ML tree.

Genus	Isolates	Characters	Informative	% Informative	Model	-lnL
*Aureobasidium*	14	517	44	8.51	SYM+I	1317.537
*Cladosporium*	36	482	45	9.34	SYM+R3	1530.217
*Filobasidium*	21	619	170	27.46	TPM2u+F+R3	3027.598
*Naganishia*	18	588	67	11.39	TIM2+F+R3	1906.687
*Papliotrema*	29	507	143	28.21	GTR+F+I+G4	2833.792
*Rhodotorula*	21	572	139	24.30	GTR+F+R3	2888.392
*Sampaiozyma*	11	553	63	11.39	TIM3+F+I+G4	1541.096
*Sporobolomyces*	34	560	151	26.96	TIM2+F+I+G4	3160.923
*Symmetrospora*	17	582	131	22.51	GTR+F+G4	2778.027
*Vishniacozyma*	25	493	157	31.85	TIM2+F+G4	2545.886

**Table 2:** All genera obtained from romaine lettuce samples in this study, with number of species, number of total isolates and number of isolates by treatment (conventional, organic, hydroponic).

Genus	Species	Total Isolates	Conventional	Organic	Hydroponic
*Acanthophysium*	1	1		1	
*Alternaria*	6	6	5		1
*Aureobasidium*	2	10	6	4	
*Beauveria*	1	1			1
*Botryotinia*	1	1	1		
*Bullera*	2	2	2		
*Bulleromyces*	1	1		1	
*Cladosporium*	8	59	19	34	6
*Cystofilobasidium*	2	12	7	5	
*Epicoccum*	1	1		1	
*Filobasidium*	6	33	16	17	
*Gibellulopsis*	1	3	2	1	
*Holtermanniella*	1	1	1		
*Leucosporidium*	1	1		1	
*Metschnikowia*	2	3	1	2	
*Moesziomyces*	1	1			1
*Mucor*	1	1		1	
*Naganishia*	1	1	1		
*Ophiostoma*	1	1		1	
*Papiliotrema*	2	3	1	2	
*Penicillium*	2	3	1	2	
*Rhodotorula*	3	3	1	1	1
*Sampaiozyma*	2	2	1		1
*Sporobolomyces*	2	67	36	31	
*Stemphylium*	1	2		2	
*Symmetrospora*	1	2		2	
*Tilletiopsis*	1	1		1	
*Vishniacozyma*	5	27	9	18	
**Total**	**59**	**249**	**110**	**128**	**11**

## Data Availability

Not applicable.

## Supplementary Materials

The following are available online at https://www.mdpi.com/article/10.3390/jof7040277/s1, Table S1: GenBank accession numbers of ex-type strains used in core community datasets.

## Appendix A

**Table A1:** Results from BLAST searches of contigs (with multiple identical isolates) or individual isolates in tabular format. For each contig or isolate, the following are shown: species name, number of isolates (total and by treatment: C, conventional; O, organic; H, hydroponic) and best match information (species name, strain information, GenBank accession number, type status, percentage of similarity and reference in which the sequence was first published). Query coverage was between 75 and 100% for all BLAST searches.

Contig or Isolate	Species	Number of Isolates				Best Match	Strain	Sequence	Similarity [%]	Reference
		All	C	O	H					
C_0008	*Sporobolomyces* sp. nov. 2	66	36	30		*Sporobolomyces roseus*	CBS:7683^T^	KY105472	98.84	[20]
C_0005	*Cladosporium* sp. 1	33	12	20	1	*Cladosporium xylophilum* (**a**)	CBS:125997^T^	MH863875	100.00	[20]
C_0016	*Filobasidium stepposum*	23	10	13		*Filobasidium stepposum*	CBS:10265^T^	NR_111207	99.83	[32]
C_0010, HU9126, HU9242	*Vishniacozyma victoriae*	16	3	13		*Vishniacozyma victoriae*	ATCC:MYA-305^T^	MH809977	99.81–100.00	R.E. Rush et al. unpubl.
C_0063	*Cystofilobasidium* aff. *macerans* sp. nov. 1	11	7	4		*Cystofilobasidium macerans*	CBS:10757^T^	KY103183	98.24	[20]
C_0009	*Cladosporium* sp. 5	10	2	8		*Cladosporium iridis* (**b**)	CBS:138.40^T^	EU167591	100.00	[154]
C_0070, HU9199	*Aureobasidium pullulans*	7	4	3		*Aureobasidium pullulans* (**c**)	CBS 584.75^T^	KT693733	100.00	[53]
C_0038, HU9045	*Vishniacozyma* cf. *foliicola*	6	3	3		*Vishniacozyma foliicola*	CBS:9920^T^	KY105821	98.67–98.86	[20]
C_0014	*Cladosporium* sp. 3	5	3	1	1	*Cladosporium puyae* (**d**)	CBS:274.80A^T^	NR_152298	100.00	[69]
C_0053, HU9127	*Filobasidium magnum*	5	2	3		*Filobasidium magnum*	CBS:140^T^	NR_130655	99.67–100.00	[34]
C_0006	*Cladosporium* sp. 2	4	2	2		*Cladosporium limoniforme* (**d**)	CBS:140484^T^	KT600397	100.00	[69]
C_0104	*Aureobasidium subglaciale*	3	2	1		*Aureobasidium subglaciale*	CBS:123387^T^	KT693735	100.00	[53]
C_0009b	*Cladosporium* sp. 4	3		3		*Cladosporium rhusicola* (**d**)	CBS:138.40^T^	NR_152299	100.00	[69]
C_0003	*Gibellulopsis nigrescens*	3	2	1		*Gibellulopsis nigrescens*	CBS:120949^T^	NR_149327	100.00	[155]
C_0001, HU9254	*Vishniacozyma carnescens*	3	2	1		*Vishniacozyma carnescens*	CBS:973^T^	KY105817	99.84–100.00	[20]
C_0224	*Cladosporium* sp. 7	2			2	*Cladosporium endophyticum*	MFLUCC:17-0599^T^	MG646956	99.11	[156]
C_0071	*Filobasidium oeirense*	2	1	1		*Filobasidium oeirense*	CBS:8681^T^	KY103438	99.37	[20]
C_0215	*Metschnikowia picachoensis*	2		2		*Metschnikowia picachoensis*	NRRL:Y-27607^T^	AY494780	100.00	[157]
C_0205	*Papiliotrema* aff. *fonsecae* sp. 1	2		2		*Papiliotrema fonsecae*	EX:F-4087^T^	NR_119972	99.76	[32]
C_0057	*Penicillium flavigenum*	2	1	1		*Penicillium flavigenum*	CBS:419.89^T^	MH862182	100.00	[158]
C_0045	*Stemphylium* sp. 1	2		2		*Stemphylium waikerieanum*	VPRI:21969^T^	MK336832	100.00	[70]
C_0048	*Symmetrospora* sp. nov.	2		2		*Symmetrospora coprosmae*	CBS:7899^T^	NR_073317	99.48	[101]
HU9265	*Acanthophysium* sp. 1	1		1		*Acanthophysium bisporum*	CBS:240.86^T^	MH861954	90.02	[158]
HU9315	*Alternaria* sp. 1	1			1	*Alternaria angustiovoidea* (**e**)	CBS:195.86^T^	MH861939	100.00	[158]
HU9181	*Alternaria* sp. 2	1	1			*Alternaria ventricosa* (**e**)	CBS:121546^T^	MH863116	100.00	[158]
HU9087	*Alternaria* sp. 3	1	1			*Alternaria dactylidicola*	MFLUCC:15-0466^T^	NR_151852	99.01	[159]
HU9178	*Alternaria* sp. 4	1	1			*Alternaria conjuncta*	CBS 196.86^T^	MH861940	99.67	[158]
HU9194	*Alternaria* sp. 5	1	1			*Alternaria eureka*	CBS 193.86^T^	MH861937	98.78	[158]
HU9226	*Alternaria* sp. 6	1	1			*Alternaria arbusti* (**e**)	CBS 596.93^T^	MH862447	100.00	[158]
HU9303	*Beauveria* cf. *bassiana*	1			1	*Beauveria bassiana*	ARSEF:1564^T^	NR_111594	98.91	[32]
HU9220	*Botryotinia pelargonii*	1	1			*Botryotinia pelargonii*	CBS:497.50^T^	AJ716290	100.00	[160]
HU9201	*Bullera* aff. *unica* sp. 1	1	1			*Bullera unica*	CBS:8290^T^	NR_073256	99.62	[32]
HU9228	*Bullera unica*	1	1			*Bullera unica*	CBS:8290^T^	NR_073256	100.00	[32]
HU9149	*Bulleromyces albus*	1		1		*Bulleromyces albus*	CBS:500^T^	KY101811	100.00	[20]
HU9304	*Cladosporium* sp. 6	1			1	*Cladosporium puyae*	CBS:274.80A^T^	NR_152298	99.82	[69]
HU9302	*Cladosporium sphaerospermum*	1			1	*Cladosporium sphaerospermum*	ATCC:11289^T^	AY361958	100.00	[161]
HU9177	*Papiliotrema frias*	1	1			*Cryptococcus frias*	CRUB 1250^T^	GU997162	100.00	[89]
HU9106	*Cystofilobasidium infirmominiatum*	1		1		*Cystofilobasidium infirmominiatum*	CBS:323^T^	NR_073232	100.00	[32]
HU9025	*Epicoccum nigrum* complex sp. 1	1		1		*Epicoccum layuense*	CGMCC: 3.18362^T^	NR_158265	100.00	[162]
HU9229	*Filobasidium* aff. *floriforme* sp. 1	1	1			*Filobasidium floriforme*	CBS:6241^T^	NR_119429	99.06	[32]
HU9055	*Filobasidium chernovii*	1	1			*Filobasidium chernovii*	CBS:8679^T^	KY103413	100.00	[20]
HU9224	*Filobasidium wieringae*	1	1			*Filobasidium wieringae*	CBS:1937^T^	NR_077105	100.00	[20]
HU9215	*Holtermanniella* sp. nov. 1	1	1			*Holtermanniella takashimae*	CBS:11174^T^	NR_137721	99.42	[163]
HU9066	*Leucosporidium yakuticum*	1		1		*Leucosporidium yakuticum*	CBS:8621^T^	NR_155332	100.00	[20]
HU9283	*Metschnikowia* cf. *rubicola*	1	1			*Metschnikowia rubicola*	NRRL:Y-6064^T^	MG050888	99.64	[164]
HU9309	*Moesziomyces aphidis*	1			1	*Moesziomyces aphidis*	CBS:517.83^T^	NR_145336	100.00	[165]
HU9011	*Mucor circinelloides* f. *circinelloides*	1		1		*Mucor circinelloides* f. *circinelloides*	CBS:195.68^T^	HQ154604	99.12	[166]
HU9043	*Naganishia* sp.	1	1			*Naganishia liquefaciens* (**f**)	CBS:968^T^	NR_073220	100.00	[32]
HU9061	*Ophiostoma* sp. 1	1		1		*Ophiostoma rufum* (**g**)	CBS:144871^T^	MH837040	100.00	[167]
HU9060	*Penicillium* sp. 1	1		1		*Penicillium hoeksii*	CBS:137776^T^	NR_137913	99.63	[168]
HU9099	*Rhodotorula* aff. *glutinis* sp. nov. 1	1		1		*Rhodotorula babjevae*	CBS:7808^T^	NR_077096	99.66	[101]
HU9231	*Rhodotorula* aff. *glutinis* sp. nov. 2	1	1			*Rhodotorula babjevae*	CBS:7808^T^	NR_077096	99.49	[101]
HU9305	*Rhodotorula* sp. nov. 3	1			1	*Rhodotorula diobovata*	CBS:6085^T^	KY104768	99.51	[20]
HU9046	*Sampaiozyma* sp. nov. 1	1	1			*Sampaiozyma ingeniosa*	CBS:4240^T^	NR_111080	97.49	[32]
HU9301	*Sampaiozyma* sp. nov. 2	1			1	*Sampaiozyma vanillica*	CBS:7404^T^	KY105310	93.94	[20]
HU9138	*Sporobolomyces* sp. nov. 1	1		1		*Sporobolomyces patagonicus*	CBS:9657^T^	KY105521	98.82	[20]
HU9240	*Tilletiopsis* aff. *pallescens* sp. nov. 1	1		1		*Tilletiopsis pallescens*	CBS 606.83^T^	NR_111216	98.49	[32]
HU9248	*Vishniacozyma* aff. *heimaeyensis* sp. nov.	1	1			*Vishniacozyma heimaeyensis*	CBS:8933^T^	NR_077070	98.63	[32]
HU9122	*Vishniacozyma tephrensis*	1		1		*Vishniacozyma tephrensis*	CBS:8935^T^	NR_144812	100.00	[169]

Footnotes: (**a**) Ex-type sequences of 18 *Cladosporium* species (*C. cladosporioides* complex) with 100% similarity. (**b**) Ex-type sequences of 16 *Cladosporium* species (*C. herbarum* complex) with 100% similarity. (**c**) Ex-type sequence of *Aureobasidium proteae* (GenBank acc. no. KT693732, CBS:111973 [53]) is also 100% similar. (**d**) Ex-type sequences of 2–3 *Cladosporium* species (*C. herbarum* complex) with 100% similarity. (**e**) Ex-type sequences of 2 *Alternaria* species with 100% similarity. (**f**) Ex-type sequence of *Naganishia albidosimilis* (GenBank acc. no. NR_077113, JCM:8843 [32]) is also 100% similar. (**g**) Ex-type sequences of 8 *Ophiostoma* species with 100% similarity.
